# Socioeconomic status and health-related quality of life after stroke: a systematic review and meta-analysis

**DOI:** 10.1186/s12955-023-02194-y

**Published:** 2023-10-25

**Authors:** Yichao A. Sun, Serah Kalpakavadi, Sarah Prior, Amanda G. Thrift, Suzanne Waddingham, Hoang Phan, Seana L. Gall

**Affiliations:** 1 Menzies Institute for Medical Research, University of Tasmania, 17 Liverpool Street, Hobart, TAS 7000 Australia; 2 Tasmanian School of Medicine, Rural Clinical School, University of Tasmania, Burnie, Australia; 3 Stroke and Ageing Research, Department of Medicine, School of Clinical Sciences at Monash Health, Monash University, Melbourne, Australia; 4 Tasmanian School of Medicine, University of Tasmania, Hobart, Australia; 5 Menzies School for Health Research, Charles Darwin University, Casuarina, Australia

**Keywords:** Stroke, Socioeconomic status, Health-related quality of life, Systematic review, Inequity, Education, Income

## Abstract

**Background:**

Socioeconomic status (SES) is associated with stroke occurrence and survival following stroke but its association with health-related quality of life (HRQoL) following stroke remains uncertain. We performed a systematic review and meta-analysis to examine the association between SES and HRQoL after stroke.

**Methods:**

PubMed, SCOPUS, EMBASE, and Web of Science were searched to identify relevant cohort and case–control studies between January 2000 and May 2022. Two authors screened titles, abstracts and full text articles. One author extracted data from all included studies. Meta-analyses were performed for studies with comparable measurements of SES and HRQoL. Random effects models were used to estimate pooled summary standardised mean differences in HRQoL by SES.

**Results:**

Out of 1,876 citations, 39 studies incorporated measurement of overall HRQoL following stroke and were included in the systematic review, with 17 studies included in the meta-analyses. Overall, reports including education, income, occupation and work status effects on HRQoL after stroke were inconsistent among all included 39 studies. In the global meta-analysis of 17 studies, HRQoL among survivors of stroke was lower in the low SES group than in the high SES group (standardised mean difference (SMD) -0.36, 95% CI -0.52, -0.20, *p* < 0.0001). When using education and income indicators separately, summary effects were similar to those of the global analysis (low versus high education SMD -0.38, 95% CI -0.57, -0.18, *p* < 0.0001; low versus high income SMD -0.39, 95% CI -0.59, -0.19, *p* < 0.0001).

**Conclusions:**

Across all SES indicators, people with stroke who have lower SES have poorer overall HRQoL than those with higher SES. Accessibility and affordability of poststroke support services should be taken into consideration when planning and delivering services to people with low SES.

**Supplementary Information:**

The online version contains supplementary material available at 10.1186/s12955-023-02194-y.

## Background

Health-related quality of life (HRQoL) may be influenced by a number of individual disease-related factors such as illness, functional status and general health perception [1]. However quality of life varies greatly despite any similarities in patients’ stroke severity and current health state [2]. In the last two decades, socioeconomic factors have also been recognised to affect HRQoL [3].

Socioeconomic status (SES) comprises a number of different aspects of an individual’s economic resources and social status, commonly including a combination of individual-level factors such as income, education and occupation and/ or area-level factors of economic resources such as housing. These markers have been frequently used as indicators, individually or in combination, to measure SES [4]. Previous literature provides evidence that socioeconomic disparities have a profound impact on stroke mortality and functional outcomes including mobility and cognition with little or no exploration of quality of life as an outcome [5].

To date, the effect of SES on HRQoL after stroke has not been systematically reviewed. There is conflicting evidence about the association between SES and HRQoL after stroke. Some investigators have reported associations between unemployment and manual occupation with poor HRQoL, [6, 7] some reporting associations between level of education and occupation limited to the physical or mental aspect of HRQoL, [8, 9] while in some reports, the evidence was equivocal [10]. With increasing recognition of the role of social determinants of health as important outcomes following stroke, [11] a systematic review of the association between SES and HRQoL will fill an important gap in our understanding of how these factors may influence the outcomes for people with stroke. Using a systematic review design, we aimed to determine the association between socioeconomic status and HRQoL in people who have had a stroke.

## Methods

We systematically identified studies undertaken to examine the association between SES and HRQoL after stroke. This review protocol has been registered in the International Prospective Register of Systematic Review (PROSPERO) with the protocol number: CRD42022336865.

### Data sources

We used the PECO framework: Population (P): people with stroke; Exposure (E): low socioeconomic status with indicators such as income, education, occupation: Comparison (C): high socioeconomic status; Outcome (O): health-related quality of life. Relevant keywords were used to build a search strategy in these databases to retrieve the appropriate publications (Supplementary Table S1). To account for spelling variations, truncation and wildcards were used when building the search strategy for each database. Four databases: PubMed, SCOPUS, EMBASE, Web of Science were used in electronic search (Supplementary Table S2). Manual search of reference lists in all included articles were also conducted.

Two reviewers (YAS and SK) with healthcare backgrounds and postgraduate qualifications in research independently screened the titles and abstracts of all retrieved studies for inclusion in full text screening. The inclusion criteria were: 1) primary cross sectional, cohort or case–control observational study; 2) SES indicator as a predictor or as a covariate; 3) all ages and settings; 4) articles published between January 2000 to May 2022; and 5) published in English language. The exclusion criteria were: 1) studies conducted in specific clinical populations such as cancer and kidney disease 2) review articles; 3) studies incorporating HRQoL as an outcome in an intervention study; 4) studies conducted to test the validity of an instrument; 5) studies among people with transient ischemia attack; and 6) qualitative studies. Studies that met the selection criteria were processed for data extraction. Discrepancies were resolved by discussion between the two reviewers. If a conflict persisted, the third reviewer (SLG), a senior epidemiologist, was consulted for the final decision. For the full text screening, the Kappa coefficient for agreement between the two initial reviewers was 0.87.

### Data extraction

Data were extracted on author, year of publication, study design, location, data source, sample size, SES indicator, HRQoL instrument, follow-up time for cohort study, mean age, sex, stroke type, whether or not the study focused on SES, HRQoL measurement in any domains, other variables included in the multivariable analyses and method of statistical analysis. All data were extracted by YAS. Corresponding authors of relevant articles were contacted for further information if required. When multiple follow-up times were reported, the longest follow-up time was extracted. For studies that qualified for the meta-analysis, we extracted additional data including sample size, mean and standard deviation (SD) for each SES group, estimates with confidence interval (CI), *P*-value, t-value.

### Quality assessment

The Joanna Briggs Institute (JBI) critical appraisal tools (cohort study, case–control study, cross-sectional study) [12] were used to assess the quality of studies for all included papers by YAS. The study was scored 1 for Yes when it met the criterion, 0 for unclear and -1 for No. A question was excluded when it was not applicable to a particular study. To make all studies comparable when assessing quality, the scores gained from assessment tools for cohort and case–control studies were then converted to proportions. Quality was considered high (85%-100%), medium (65%-84) and low (below 65%). Uncertainties were discussed with the third reviewer (SLG).

### Data synthesis and statistical analysis

We initially included all eligible studies of SES and HRQoL after stroke. For qualitative synthesis, we then limited the studies to those with outcomes of overall HRQoL. For the meta-analysis, we only included studies that incorporated use of overall HRQoL such as an overall quality of life score or a utility score. This included studies that had a similar construction of instruments for assessing HRQoL with multi-domains including physical, mental and social aspects.

We conducted a ‘global’ meta-analysis combining studies across different SES indicators. We examined the data source and study period to identify potentially overlapping population. If overlapping studies were found, we used the JBI tool to select the highest quality study for the ‘global’ meta-analysis. If the JBI score was the same, we selected the studies with larger study population then the most recent study. In the global meta-analysis, if multiple SES indicators were used in the one study, we prioritised SES indicators to those with an SES index—an indicator that includes multiple SES attributes, and then to individual-level SES markers in the following order: income, education level, occupation, work status and others. Because there is no individual best SES indicator, we prioritised income because it is suggested to be the optimal indicator for better material advantage and service access [13] and has been shown to be the most important indicator in the adult population in health research [14, 15]. If original data with number, mean and standard deviation (SD) of each group were available, and if more than two SES groups were reported, the groups were combined to generate medium/high and low/medium SES groups. Calculation of combined mean and SD was based on methods described by the Cochrane Collaboration documentation [16]. If the original data were not available, the pre-calculated effect size data were used. In these instances, if more than two SES groups were reported, the highest and the lowest SES groups were extracted. When multiple models were reported, we selected the model that controlled for at least age and sex, these being the most important potential confounders, as well as allowing for consistency across studies. Given the heterogeneity of HRQoL measures, standardized mean differences (SMD) were used in the meta-analyses to enable comparison among studies with diverse HRQoL instruments. Analyses were repeated for each individual SES indicator across all available studies.

Sensitivity analyses were performed to exclude studies with a higher risk of bias. We conducted leave-one-out influence analysis to assess the outlier and repeated the meta-analysis without the potential influence study. A random-effect model was used to account for the heterogeneity between studies. Two-sided *P*-value ≤ 0.05 was considered as statistically significant. Heterogeneity was assessed using the Q statistic, I^2^ statistic and τ. We examined publication bias or small-study effects by inspecting funnel plots and using Egger’s test. For sub-group analyses, we stratified the studies by the study-level factors: adjusting for confounders, stroke type, and whether the study was designed to specifically investigate SES and study region. All statistical analyses and graphics were performed using R 4.1.2.

## Results

### Literature search

**Fig. 1:**
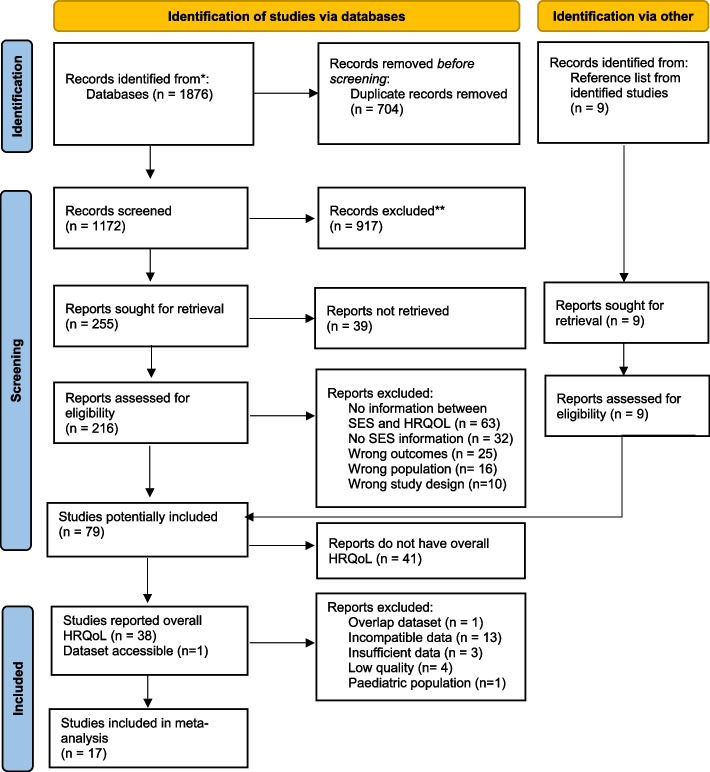
PRISMA 2020 flow diagram for new systematic reviews which included searches of databases and manually search reference list. *From:* Page MJ, McKenzie JE, Bossuyt PM, Boutron I, Hoffmann TC, Mulrow CD, et al. The PRISMA 2020 statement: an updated guideline for reporting systematic reviews. BMJ 2021;372:n71. https://doi.org/10.1136/bmj.n71. For more information, visit: http://www.prisma-statement. *Records identified from PubMed, SCOPUS, EMBASE and Web of Science. **Records excluded after screening titles and abstracts

We identified 1,876 potential articles for inclusion (Fig. 1). After excluding 704 duplicate records, 1172 records were screened by title and abstract, and 255 records were selected for full-text screening. Seventy articles were deemed relevant. A further nine articles were identified following screening of references from relevant studies resulting in a total of 79 articles meeting the inclusion criteria. The overall HRQoL outcomes were reported in 38 out of 79 studies, and one in the remaining 41 studies has the online dataset available to access.

### Study characteristics

Among the 39 publications included, most included all stroke types, and education was the most commonly utilised SES indicator (Table 1). Only two studies incorporated SES indices, one being an area-level SES index and one a paediatric four-factor index of social status. Most included studies (97%) in the systematic review measured SES at individual level, such as income, education and employment status, with only 3% measured SES at area level. Among all 39 studies, most studies incorporated more than one SES indicator. A total of 17 different instruments were used to assess overall HRQoL (Supplementary Table 3). Various versions of one type of instrument were used. The WHOQOL was the most frequently used instrument followed by the Stroke Specific QoL (SS-QoL) and the EQ-5D assessment (Supplementary Table 3). Most studies were a cross-sectional design or provided estimates in cross-sectional analysis. While studies were conducted worldwide, Korea had the greatest number of individual studies. The majority studies were focused on the determinants of HRQoL after stroke, while only three were specifically focussed on the associations between SES and HRQoL. English versions of HRQoL instruments were translated and validated in Non-English speaking countries. Approximately, 80% studies had a low risk of bias.

**Table 1:** Summary of studies included in the systematic review (*n* = 39)

Author	Year	Study design	Location	Data source	n	Socio-economic indicator						Instrument	Stroke type	Focused on SES
						**SES index**	**income**	**education**	**occupation**	**work status**	**others**			
Abubakar [17]	2012	cross sectional	Nigeria	hospital based	62			X				SIS-16	All	no
Ali [18]	2017	cross sectional	Iraq	outpatient department	80			X	X		X	SS-QoL-12	All	no
Alshahrani [19]	2020	cross sectional	Saudi Arabia	hospital based	123		X	X		X	X	WHOQOL-BREF	All	other †
Barbosa [20]*	2022	Longitudinal §	Portugal	hospital based	349		X	X		X		SF6D/EQ-5D-3L	All	other
Baune [21]*	2006	case–control	Gaza Strip	outpatient department	112		X	X				WHOQOL-BREF	All	no
Butsing [22]*	2019	cross sectional	Thailand	in-patient and outpatient department	358		X	X		X		WHOQOL-BREF	All	no
Choi-Kwon [23]*	2006	cross sectional	Korea	outpatient department	151		X	X		X		WHOQOL-100	All	other
Chou [24]	2015	cross sectional	Taiwan	hospital based	134			X		X		SS-QoL-12	All	other
Chuluunbaatar [25]	2016	Longitudinal §	Mongolia	hospital based	155			X				WHOQOL-BREF	All	other
Cramm [26]	2012	cross sectional	Netherlands	hospital based	211			X				EQ-5D	All	no
Dayapoglu [27]	2010	cross sectional	Turkey	outpatient department	70			X		X	X	SF-36	All	other
Delcourt [28]	2011	cross sectional	China	ChinaQUEST, hospital based	6427		X	X		X	X	QOL-35	All	other
Dhamoon [10]	2010	longitudinal	US	NOMAS, population-based	525			X			X	QLI-Spitzer	IS	other
Dianati [29]*	2021	cross sectional	Iran	hospital based	188		X	X				SIS-16	All	other
Ghotra [30]	2018	cross sectional	Canada	hospital registry	59	X						PedsQL4.0	IS	other
Gurcay [31]	2009	cross sectional	Turkey	in-patient and outpatient department	67			X				SIS-16	All	other
Heiberg [32]	2020	longitudinal	Norway, Denmark	stroke registries	304			X		X		QOLIBRI-OS	All	other
Huang [33]	2010	cross sectional	Taiwan	outpatient department	102			X				QLI-Ferrans	IS	other
Jun [34]*	2015	cross sectional	Korea	Korean Community Health Survey	4604		X	X	X		X	EQ-5D	All	yes
Kariyawasam [9]*	2020	cross sectional	Sri Lanka	hospital based	257		X	X	X			SAQOL-39	All	other
Kim [35]*	2021	cross sectional	Korea	outpatient department	170		X	X		X		SS-QoL-12 K	IS	no
Lee [36]	2015	case–control	Korea	hospital based	30		X					SAQOL-39	All	other
Lourenço [37]	2021	cross sectional	Portugal	outpatient department	102		X	X		X		WHOQOL-BREF	All	other
Mei [38]*	2022	cross sectional	China	Henan Rural Cohort	1709		X	X				EQ-5D-3L	All	other
Meyer [39]*	2010	Longitudinal §	Germany	University based	94		X	X				EQ-5D	HS	other
Vincent-Onabajo [40]	2015	cross sectional	Nigeria	hospital based	55					X		HROoLISP-40	All	other
Ones [41]	2005	cross sectional	Unknown	hospital based	88			X				NHP	All	other
Paul [6]*	2005	cross sectional	Australia	NEMESIS, population-based	978				X			AQoL	All	other
Pedersen [42]*	2021	cross sectional	Norway and Denmark	outpatient department	369					X		SS-QoL	All	no
Pucciarelli [43]	2019	longitudinal	Italy	hospital based (2015–2017)	405			X		X		WHOQOL-BREF	All	other
Ramos-Lima [44]	2018	cross sectional	Brazil	outpatient department	131		X	X				SS-QoL	IS	other
Salehi [45]*	2019	longitudinal	Iran	hospital based	172		X	X	X			SIS -16	All	other
Singhpoo [46]	2012	cross sectional	Thailand	hospital based	237		X	X		X		SF-36	All	other
Sok [47]*	2021	cross sectional	Korea	hospital based	146		X	X		X		SS-QoL	All	no
Sturm [48]	2004	cross sectional	Australia	NEMESIS, population-based	225				X			AQoL	All	other
Szocs [49]*	2020	cross sectional	Europe	EuroHOPE, stroke center	108/87 X		X	X		X		EQ-5D-5L	IS	yes
Taufique [50]	2016	Longitudinal §	US	the Columbia University SAH Oucomes Project	1181			X				SIP	HS	other
Tsalta-Mladenow [51]*	2021	Longitudinal §	Bulgaria	hospital based	143			X		X		SIS 3.0	IS	yes
Zemed [8]*‡	2021	cross sectional	Ethiopia	outpatient department	180		X	X	X			RAND 36-Item Health Survey	All	other

See Supplementary Table 3 for an explanation of all abbreviations

^*^Studies were included in the meta-analysis

^†^Other factors such as social support, healthcare and health behaviour were focused in the studies

^‡^Dataset is available to generate overall HRQoL score

^§^Longitudinal study with cross sectional analysis

^‡^Dataset is available to generate overall HRQoL score

### Qualitative synthesis

#### Education

Overall, the reporting of education level and HRQoL was inconsistent across the studies. Most investigators measured level of education or years of education. In most studies, a higher level of education was significantly associated with greater HRQoL in univariable analyses, but this association was attenuated after adjusting for other factors (Supplementary Table 4). In only four studies were the associations between education and HRQoL consistent for both unadjusted and adjusted analyses, while three did not include the specific magnitude of effect for nonsignificant findings in the final model.

#### Income

Income was measured most often by individual or household monthly income (*n* = 11 studies) but variations existed, including sufficiency of income (*n* = 1 study) and with or without income (*n* = 1 study; Supplementary Table 5). In three studies the measure used to classify income was not specified. The association between greater income and greater overall HRQoL was mostly consistent, with 11 having significant associations in unadjusted analysis and seven studies in adjusted analyses.

#### Occupation and work status

Definitions of occupation and work status were variable. Most studies defined occupation based on the type of job and only one study incorporated use of a local national standard of occupation which was based on skill level (Supplementary Table 6). The timing of work status also varied, with most (*n* = 22) being focussed on work status after stroke and the remainder on working prior to the stroke (*n* = 3). Overall, there was lack of clear definition of occupation and work status. In seven of the 25 studies there was a significant association between occupation or work status and overall HRQoL in adjusted analyses. In one of these studies, where current work status, as opposed to work status prior to stroke, was the SES indicator, there was a significant association in both unadjusted and adjusted results.

#### Other SES indicators

Both residency and health insurance were also used as SES indicators, with only three of eight studies providing evidence of an association between these markers and overall HRQoL (Supplementary Table 7). In one of these studies people with Medicaid had better HRQoL trajectory up to 5 years after their stroke than those without Medicaid. In another study conducted in a paediatric population that incorporated use of an SES index high SES was associated with better HRQoL [30]. Another study in which a neighbourhood SES indicator was used, there was no evidence that neighbourhood SES was independently associated with overall HRQoL [49].

#### Meta-analysis of overall HRQoL

Among 39 studies (38 studies in which overall HRQoL was reported and one [8] in which overall HRQoL was calculated from raw data), 13 studies had incompatible data, one had an overlapping dataset, and three had insufficient data. This left a total of 22 studies eligible for meta-analysis. After excluding four studies with a high risk of bias and a study on a paediatric population, we included 17 studies with a total number of 8,332 subjects in the global meta-analysis.

#### Global meta-analysis of HRQoL

**Fig. 2:**
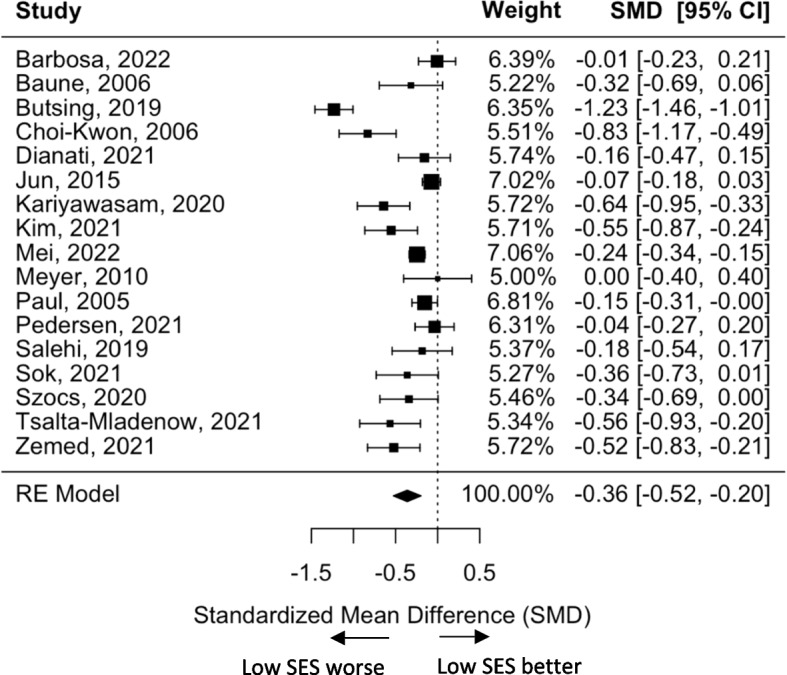
Standardized mean differences and 95% CIs of the low vs high SES from global meta-analysis using combined SES indicators and overall HRQoL (*n* = 17 studies)

Overall, HRQoL was less in the low SES group than in the high SES group with an SMD of -0.36 (95% CI -0.52, -0.20, *p* < 0.0001; Fig. 2). However, the effects across all studies were inconsistent, ranging from 0 to -1.23, and a large heterogeneity (Q = 121.5, I^2^ = 88.4%, τ = 0.3). Using the leave-one-out analysis the precision of the model was largely influenced by the study by Butsing et al. [22]. After excluding this study, the heterogeneity was reduced (Q = 47.7, I^2^ = 75%, τ = 0.2) and there was a slight reduction in the SMD to -0.29 (95% CI -0.41, -0.17). The funnel plot (Supplementary Fig. 1) among the 17 studies was symmetric with the Egger’s test providing *P* = 0.44, suggesting that no potential publication bias or small-study effects were found.

#### Meta-analysis by SES indicators

When examining income and education indicators separately, the effects were similar, with SMD -0.39 for income and -0.38 for education, both having large heterogeneity (Tables 2 and 3). The Egger’s test for income (*P* = 0.77) and education (*P* = 0.40) meta-analyses also provided evidence for no potential publication bias.

**Table 2:** Studies included in the meta-analysis (*N* = 17)

Author (Year)	N^a^	Income		Education		Occupation		Work status		Adjusting factors^d^	Raw^b^	Pre-calculator^c^
		**high**	**low**	**high**	**low**	**high**	**low**	**high**	**low**			
Barbosa (2022) [20]	349	> 1,000	< 1,000	high school	elementary					age, gender, monthly income, residence, occupation, NIHSS, mRS, BI, MMSE, length of stay, door-to-neurological examination time, access, frequency and satisfaction with rehabilitation care		X
Baune (2006) [21]	112	high	low	> = secondary school	< secondary school					none	X	
Butsing (2019) [22]	358	sufficient with saving	Insufficient/ sufficient							none	X	
Choi-Kwon (2006) [23]	151	high	poor/ moderate	> = 11 years	< 11 years					none	X	
Dianati (2021) [29]	188	good	poor/ moderate	diploma/ university	illiterate/ primary					none	X	
Kariyawasam (2020) [9]	257	> 10,000	< 10,000	advanced	up to ordinary					none	X	
Kim (2021) [35]	170	> = 200	< 200	high school/ college	middle/ elementary school					none	X	
Jun (2015) [34]	2847	301–400	< = 100	> = high school	< = elementary					Age, sex, education, income, occupation, residential area, living with family		X
Mei (2022) [38]	1709	> 143	< 72	other	illiterate/ primary					none	X	
Meyer (2010) [39]	94	> 1,000	< 1,000	> = 12 years	< 12 years					none	X	
Paul (2005) [6]	978					nonmanual	manual			age, hemiplegia, initial NIHSS score		X
Pedersen (2021) [42]	369							working/ student	Sick/ retired	age, gender, marital status, independent, stroke severity, stroke type, length of stay, anxiety, depression		X
Salehi (2019) [45]	172	with income	no income							none	X	
Sok (2021) [47]	146	> = 200	< 200	high school/ college	middle/ elementary school					none	X	
Szocs (2020) [49]	109	SES index: deprived/no deprived area								none	X	
Tsalta-Mladenow (2021) [51]	143			tertiary	primary/ secondary					none	X	
Zemed (2021) [8]	180	> 4,000	< 4,000	> = grade 9	< = grade 8					none	X	

*NIHSS* National Institutes of Health Stroke Scale, *mRS* Modified rankin scale, *BI* Barthel Index, *MMSE* Mini-Mental State Examination

^a^N is the number that in groups included in the meta-analysis

^b^Data are the number, mean and standard deviation used calculate effect size

^c^Regression coefficient or p-value or F statistics were used to calculate the effect size

^d^Factors adjusted for in analysis that are incorporated in the meta-analysis

**Table 3:** Meta-analysis by indicators of SES and their association with HRQoL among people with stroke from all included studies

SES Indicator	n	Estimate	95% CI	*P*	Q	I^2^	τ
Global analysis	17	-0.36	-0.52, -0.20	< 0.0001	121.5	88.4%	0.3
Income only	13	-0.39	-0.59, -0.19	< 0.0001	112.4	90.1%	0.1
Education only	13	-0.38	-0.57, -0.18	< 0.0001	67.0	89.3%	0.3

#### Sub-group analyses

In the ‘global’ meta-analysis, the estimated SMD was 0.38 less for studies with confounder adjustment than in those without adjustment but remained statistically significant (Table 4). Age, sex, stroke severity and disability were the most common variables incorporated within the multivariable models. The test for sub-group differences was statistically significant between unadjusted and adjusted study groups as well as between hospital and community settings (Table 4). There was no evidence for a sub-group effect according to stroke type, purpose (specifically undertaken to investigate associations between SES and HRQoL versus not), or SES region. In sub-group analyses conducted separately for income and education indicators which were stratified by adjustment for potential confounders, significant sub-group effects were observed in both groups, with large heterogeneity observed in the unadjusted models (Supplementary Table 8). In the studies that had fully adjusted models, we were unable to detect an association between income and HRQoL (Supplementary Table 8).

**Table 4:** Association between SES and HRQoL of studies included in the global analysis: sub- group analyses

Sub-group analysis	n	Estimates	95% CI	*p*	I^2^	*P* for subgroup
**Adjustment**^a^						** < 0.001**
Adjustment	4	-0.08	-0.16, -0.01	0.03	0	
Non-adjustment	13	-0.46	-0.65, -0.28	< 0.0001	82.4	
**Type of stroke**						0.099
All	13	-0.36	-0.56, -0.16	0.0004	91.6	
Ischemic	3	-0.48	-0.68, -0.29	< 0.0001	0	
Haemorrhagic	1	0	-0.40, 0.40			
**Study designed specifically to investigate SES and HRQoL**						
No	6	-0.42	-0.79, -0.04	0.028	91.0	0.860
Yes	3	-0.29	-0.58, 0.01	0.059	72.7	
Predictors	8	-0.33	-0.52, -0.15	0.0004	78.8	
**Population age (mean, years)**						
> 65	11	-0.35	-0.58, -0.13	0.002	89.5	0.982
≤ 65	6	-0.38	-0.59, -0.17	< 0.001	68.7	
**Follow-up time (mean, month)**						
< 3	5	-0.38	-0.58, -0.18	< 0.001	42.8	0.392
3–12	4	-0.14	-0.40, 0.11	0.27	69.8	
> 12	2	-0.48	-1.14, 0.19	0.16	92.2	
Not available	6	-0.46	-0.79, -0.12	0.008	95.1	
**Study year**						0.119
Before 2016	9	-0.23	-0.38, -0.09	< 0.001	76.6	
2016 and later	8	-0.49	-0.76, -0.21	< 0.001	86.2	
**Study setting**						**0.024**
Hospital/Clinic	14	-0.41	-0.61, -0.22	< 0.001	81.7	
Community	3	-0.16	-0.27, -0.05	0.003	61.3	
**SES region**^b^						0.929
High – upper middle	12	-0.36	-0.58, -0.14	0.0011	92.6	
Low – lower middle	5	-0.37	-0.57, -0.18	0.0002	41.9	

^a^Models are fully adjusted for potential confounders

^b^SES region was classified based on World Bank income level 2021 of the study location

## Discussion

In this systematic review we identify that reports on the associations between different markers of SES, such as income or education level, and overall HRQoL vary across studies. Based on general rule of interpretation of SMD, SMD < 0.2 is a “small” effect, between 0.2–0.5 is a “moderate” effect and > 0.8 is a “large” effect [52]. Our findings demonstrated a moderate effect of low SES, such as low education level and low income on HRQoL among stroke survivors. Interestingly, in the meta-analysis, there was consistent evidence that exposure to low SES, compared to high SES, was associated with a poorer overall HRQoL after stroke regardless of SES indicator used.

Different SES indicators, such as education, income and a ‘global’ analysis using mixed indicators, have similar associations with HRQoL after stroke, supporting the notion that any aspect of SES could affect HRQoL after stroke. This study extends previous knowledge of the association between SES and stroke incidence and mortality [5]. The findings suggest that SES should be considered as an important determinant of HRQoL, which is a key patient-reported outcome measure, in both acute and subacute settings. Our findings show that any SES indicator such as income and education have similar effects on people’s HRQoL after stroke. This is supported by our observation that SMD estimates and 95% CIs were similar across the global meta-analysis and each of the individual meta-analyses. It is widely acknowledged that income and education are moderately correlated [53]. Low income or low paying jobs could limit the ability of stroke survivors to access fee-for-service treatments, thereby affecting recovery and HRQoL. A person’s income and education can affect their knowledge, attitudes, beliefs and access to healthy food, health services, health literacy, lifestyle, and environments, such as safe neighbourhood [54]. Our findings suggest that an interplay of income, education and occupation may have similar influences in an individual’s physical, mental and social health measured through HRQoL after stroke.

Few studies, < 10%, were specifically designed to examine the associations between SES and HRQoL after stroke so there may be some selective reporting. This is because in studies not designed to examine SES and HRQoL investigators may not report findings for SES indicators if no statistically significant association was found in univariable analyses. Similarly, many investigators do not report the magnitude of the association between SES and HRQoL in multivariable models because the SES effect is no longer statistically significant. The lack of statistical significance of SES in this context may reflect mediation by other variables in the model, such as clinical factors or co-morbidities. This was shown in the results that after adjusted for potential confounders, the pooled estimate dramatically reduced from -0.46 to -0.08. Studies designed to examine SES differences in HRQoL after stroke are needed to provide the most robust approach, particularly with further exploration of other factors that potentially mediate the association between SES and HRQoL after stroke. Findings from these studies may be useful for developing interventions to address these differences in HRQoL by SES.

The mechanisms for the association between SES and HRQoL after stroke are likely to be complex. It is known that lower SES affects HRQoL. A person’s individual and community resources may play a role in an individual’s physical, mental, and social health measured through HRQoL after stroke. In countries with access to universal healthcare, free education and job markets, disparities in HRQoL after stroke persist [6, 49]. HRQoL may also depend on the sense of control over health, which has been suggested to differ according to SES [55, 56]. This sense of control over health is influenced by factors over the life course that are shaped by social factors including SES. These factors shape one’s understanding of people’s circumstances and health behaviours including responses to stress and adaptation of lifestyle, which may ultimately influence HRQoL during recovery from recovery.

As both HRQoL and SES are multifaceted and dynamic over the life course potential interventions are complex. It is critical to recognise the structural determinants of health inequality that may lead to this association observed in HRQoL after stroke. The unequal distribution of resources can lead to one particular social group being more vulnerable than the others across the life course. Appreciating the influence of SES over the life course can help us to understand how exposure to low SES may result in lower HRQoL after stroke. For example, to raise a person’s education level is subject to not only their education opportunities, individual circumstances such as family culture and family circumstances, but also social mobility in society. Stroke survivors with low SES may be trapped in “the cycle of deprivation” that is challenging to break to improve their HRQoL.

Our study has several implications. Because education, income and occupation can have similar effects on poststroke HRQoL, strategies that addressing education barriers would improve the access to better paying jobs and therefore increase income level. Our findings provide insights to stroke services, particularly, how community-based rehabilitation support and outpatient clinic could effectively approach to more vulnerable groups. Service planning and delivery should address potential barriers, including improving health literature, such as using plain language, to people with low education level, and providing low-fee or fee-free access for stroke survivors with low income.

There are also implications in research. As only three out of 39 studies were specifically focused on assessing the association between SES and HRQoL, the role of potentially confounding factors is not clear. Within these three SES focused studies, two adjusted for stroke severity. As stroke severity and post-stroke disability are likely to influence HRQoL and limited evidence was currently available, more studies with a focus on SES and HRQoL with the adjustment for stroke severity and clinical factors are needed. Most included studies have excluded patients who were unable to consent, failed to complete the HRQoL assessment, or patients with severe impairment such as aphasia or cognitive impairment. These populations who are likely to have different levels of HRQoL post-stroke to groups currently included should be included in future HRQoL studies [57]. Although a few studies obtained HRQoL assessment from a person’s primary caregivers, the assessment outcomes from these proxies may differ from direct assessment from the people themselves. It remains challenging to eliminate this kind of selection bias in patient reported outcomes, however, careful investigation of the reasons for people not participating in the evaluation should be included in future studies, along with efforts to use inclusive ways of assessing HRQoL (e.g. aphasia friendly HRQoL tools) to minimise the impact of such bias. In addition, SES can change after a stroke, particularly for measures of income and employment status due to the effects of stroke. Most studies assessed income level and employment status prior to stroke, only one study [23] specified that SES was measured by current employment status during the interview after stroke. However, the change of SES in relation to HRQoL was not examined in that study. Future studies could also explore how the change of SES before and after stroke affects HRQoL.

Several limitations of this study should be noted. We did not conduct a sub-group analysis with occupation because of variations in measurement. For example, occupation was classified by skill level, [6] currently working or not, [20, 47, 51] or based on characteristics of employment. [8, 9] Only five of the 18 studies with adjustment for potentially important confounders could be included in the meta-analysis. As unadjusted results in observational studies are more likely to be biased, the summary effects of the association combining unadjusted and adjusted estimates may be overestimated. Sub-group analysis with only adjusted studies showed an overall smaller effect size but remained significant. Additionally, the unexplained heterogeneity remained large despite the sub-group analyses being stratified by SES indicators. Factors such as measurement of SES, timepoint of HRQoL assessment post stroke and characteristics of the study populations may have contributed to the heterogeneity. HRQoL is subjective, dynamic and multidimensional, so instruments combined across studies may not reflect all aspects of quality of life, particularly psychological and spiritual aspects. Data extraction and quality assessment were conducted by one reviewer and there might be errors or misclassifications. We minimised this risk by using pre-defined rules in data extraction and quality assessment. In meta-analyses, we have combined high/medium to high SES and medium/low to low SES groups when using the raw data from primary studies. The simplification of these grouping may have lost some precision and richness of the data and potentially some nuance in how different levels of SES is associated with HRQoL. In general, the exposure of low or high SES may generally represent the below average and above average SES populations. We only included studies that incorporated measurement of overall HRQoL, excluding 41 studies having subdomains of HRQoL. SES indicators may be associated with some subdomains of HRQoL, and this association would be missed in this review. Finally, we restricted our review to peer reviewed English publications, which is likely to have excluded some studies from non-English speaking countries. However, the ‘global’ meta-analysis included more than 14 regions showing a good representation of regions across the world.

In this systematic review and meta-analysis, we demonstrate that people in low SES groups had an overall poorer HRQoL after stroke compared with those from high SES group regardless of which SES indicator was used. Future studies exploring the dynamic nature of SES and HRQoL before and after stroke, as well as the factors contributing to lower HRQoL after stroke in people experiencing lower SES, may provide insights into interventions to reduce these differences.

### Supplementary Information

**Additional file 1: Supplementary Table 1. **Key words used in building search strategies. **Supplementary Table 2.** Search strategies used all databases up to May 2022. **Supplementary Table 3.** Explanation of the abbreviations in Table 1. **Supplementary Table 4.** Association between education and Health-Related Quality of Life among people with stroke: individual study results. **Supplementary Table 5.** Association between income indicator and Health-Related Quality of Life among people with stroke: individual study results. **Supplementary Table 6.** Association between occupation/work status and Health-Related Quality of Life among people with stroke: individual study results. **Supplementary Table 7.** Results by other socioeconomic status indicators and HRQoL among people with stroke: individual study results. **Supplementary Table 8.** Sub-group analysis by indicators of SES and their associations with HRQoL with adjustment and non-adjustment of potential confounders. **Supplementary Figure 1**. Funnel plot for the global meta-analysis using combined SES indicators and overall HRQoL (*n*=17 studies). 

## Data Availability

The datasets are available from the corresponding author where the data requested are considered and relevant to the study.
