# Supplementary material for: Socioeconomic status and health-related quality of life after stroke: a systematic review and meta-analysis
Source: Health Qual Life Outcomes. 2023 Oct 25;21:115. doi: 10.1186/s12955-023-02194-y (PMC10599023; doi:10.1186/s12955-023-02194-y)
Supplement: Supplementary file 1 — Additional file 1: Supplementary Table 1. Key words used in building search strategies. Supplementary Table 2. Search strategies used all databases up to May 2022. Supplementary Table 3. Explanation of the abbreviations in Table 1. Supplementary Table 4. Association between education and Health-Related Quality of Life among people with stroke: individual study results. Supplementary Table 5. Association between income indicator and Health-Related Quality of Life among people with stroke: individual study results. Supplementary Table 6. Association between occupation/work status and Health-Related Quality of Life among people with stroke: individual study results. Supplementary Table 7. Results by other socioeconomic status indicators and HRQoL among people with stroke: individual study results. Supplementary Table 8. Sub-group analysis by indicators of SES and their associations with HRQoL with adjustment and non-adjustment of potential confounders. Supplementary Figure 1. Funnel plot for the global meta-analysis using combined SES indicators and overall HRQoL (n=17 studies). [file 12955_2023_2194_MOESM1_ESM.docx]

Supplementary Table 1: Key words used in building search strategies

| **Population (P)** |  | **Exposure (E)** |  | **Outcome (O)** |
| --- | --- | --- | --- | --- |
| stroke    OR | AND | Socioeconomic status/ socioeconomic*  OR | AND | quality of life  OR |
| cerebrovascular accident  OR |  | poverty  OR |  | health-related quality of life  OR |
| cerebral h[a]emorrhage  OR |  | depriv*  OR |  | wellbeing*  OR |
| subarachnoid h[a]emorrhage |  | income*/education*/occupation* |  | life satisfaction |

Supplementary Table 2: Search strategies used all databases up to May 2022

| **Database** | **Search Strategy** | **Hits** |
| --- | --- | --- |
| PubMed | (((((stroke[MeSH Terms] AND (english[Filter])) OR (cerebrovascular accident[MeSH Terms] AND (english[Filter]))) OR (cerebral hemorrhage[MeSH Terms] AND (english[Filter]))) OR (subarachnoid hemorrhage[MeSH Terms] AND (english[Filter])) AND (english[Filter])) AND ((((((socioeconomic status[MeSH Terms] AND (english[Filter])) OR (occupation*[Title/Abstract] AND (english[Filter]))) OR (education*[Title/Abstract] AND (english[Filter]))) OR (income*[Title/Abstract] AND (english[Filter]))) OR (depriv*[Title/Abstract] AND (english[Filter]))) OR (poverty[Title/Abstract] AND (english[Filter])) AND (english[Filter]))) AND ((((quality of life[MeSH Terms] AND (english[Filter])) OR (health-related quality of life[MeSH Terms] AND (english[Filter]))) OR (wellbeing*[Title/Abstract] AND (english[Filter]))) OR (life satisfaction*[Title/Abstract] AND (english[Filter])) AND (english[Filter])) | 383 |
| Scopus | ( TITLE-ABS-KEY ( stroke  OR  "cerebrovascular accident"  OR  "cerebral hemorrhage"  OR  "subarachnoid hemorrhage" )  AND  TITLE-ABS-KEY ( "socioeconomic status"  OR  socioeconomic  OR  poverty  OR  deprivation  OR  income  OR  occupation  OR  education )  AND  TITLE-ABS-KEY ( "health-related quality of life"  OR  "quality of life"  OR  wellbeing  OR  "life satisfaction" )  AND  TITLE-ABS-KEY ( "cross sectional"  OR  longitudinal  OR  cohort ) ) | 460 |
| Web of Science | **((((AB=(stroke )) OR AB=(cerebrovascular accident )) OR AB=(cerebral h?emorrhage)) OR AB=(subarachnoid h?emorrhage))**  AND (((((((AB=(socioeconomic status)) OR AB=(social)) OR AB=(poverty)) OR AB=(depriv*)) OR AB=(income*)) OR AB=(education*)) OR AB=(occupation*)) AND (**(((AB=(quality of life)) OR AB=(health-related quality of life)) OR AB=(wellbeing*)) OR AB=(life satisfaction))AND (((TS=(cohort)) OR TS=(cross sectional)) OR TS=(longitudinal ))** | 394 |
| EMBASE | (((stroke or cerebrovascular accident or cerebral h?emorrhage or subarachnoid h?emorrhage).ti.) AND ((socioeconomic status or socioeconomic or poverty or depriv* or income* or education* or occupation*).ab.) AND ((quality of life or health-related quality of life or wellbeing* or life satisfaction).ti.))) OR ((stroke or cerebrovascular accident or cerebral h?emorrhage or subarachnoid h?emorrhage).ab.) AND ((socioeconomic status or socioeconomic or poverty or depriv* or income* or education* or occupation*).ab.) AND ((quality of life or health-related quality of life or wellbeing* or life satisfaction).ab.) AND ((cross sectional or  longitudinal  or  cohort).ab.)) | 639 |
| **Total** |  | **1876** |

Supplementary Table 3: Explanation of the abbreviations in Table 1

| **Category** | **Abbreviation** | **Explanation** |
| --- | --- | --- |
| Data source | AVAIL | the Adherence eValuation After Ischemic stroke-longitudinal Registry |
|  | CERISE | the Collaborative Evaluation of Rehabilitation in Stroke across Europe project |
|  | ChinaQUEST | the quality Evaluation of Stroke Care and Treatment study |
|  | CSHA-2 | the Canadian Study of health and Aging, the second wave of the study |
|  | EuroHOPE | the European Health Care Outcomes, Performance and Efficiency project in 6 European countries (Italy, Finland, Sweden, Scotland, the Netherlands, Hungary) |
|  | KNHANES | the Korea National Health and Nutrition Examination Survey |
|  | MEPS | the Medical Expenditure Panel Survey |
|  | NEMESIS | the North-East Melbourne Stroke Incidence Study |
|  | NHATS | the National Health and Aging Trends Study |
|  | NOMAS | the Northern Manhattan Study |
|  | REGARDS | Reasons for Geographic and Racial Differences in Stroke |
|  |  |  |
| Instruments | | |
|  | AQoL | Assessment of Quality of Life, 5 subscales: independent living, social relationships, physical senses, psychological well-being, illness |
|  | EQ-15D | 15 domains: breathing, mental function, speech, vision, mobility, usual activity, vitality, hearing, eating, elimination, sleeping, distress, discomfort and symptoms, depression and sexual activity |
|  | EQ-5D-3L/5L | 5 domains: mobility, self-care, usual activities, pain/discomfort, anxiety/depression (each with 3 levels: no problems, some problems, extreme problems; or 5 levels: no problems, slight problems, moderate problems, severe problems, extreme problems) |
|  | HRQOLISP-40 * | HRQOL in stroke patients, physical, psychological, cognitive, ecosocial, spiritual interaction, soul, spirit * soul and spirit were not included in the study |
|  | | |
|  | | |
| (Continues with next page) | | |
| **Category** | **Abbreviation** | **Explanation** |
|  | HRQOLISP-40 * | HRQOL in stroke patients, physical, psychological, cognitive, ecosocial, spiritual interaction, soul, spirit * soul and spirit were not included in the study |
|  | NHP | Nottingham Health Profile, part I 6 dimensions: sleep status, level of energy, emotional status, social isolation status, physical mobility, pain; part II work life, household tasks, social life, hobbies and interests, vocational activities |
|  | PedsQL 4.0 | 4 areas of functioning: physical, emotional, social, and school |
|  | QLI-Ferrans | the Ferrans & Powers Quality of Life Index, 38-items, measures satisfaction and importance in the physical component summary and the mental component summary |
|  | QLI-Spitzer | 5 domains: activity, daily living, health, support, outlook |
|  | QOL-35 | 35-item Chinese Quality-of-life questionnaire, developed and adapted to the Chinese culture using questions from the World Health Organization 100-item QOL Instrument and the Medical Outcomes Study 36-item Short Form Questionnaire. It has 6 domains: general, physical, independence, psychological, social, environmental |
|  | QOLIBRI-OS | the quality of life after brain injury overall scale, 6-item: physical condition, cognition emotions, ability to perform daily activities, personal and social life, current situation and future prospects |
|  | SAQOL-39 | Stroke and Aphasia Quality of Life Scale - general, 3 domains: physical, psychosocial, communication |
|  | SF-36 | 8 domains: physical functioning, role limitations due to physical problems, bodily pain, general health perceptions, vitality, social functioning, role limitations due to emotional problems, emotional well-being |
|  | SIP | the Sickness Impact Profile, 12 categories of function and 3 summary scores: psychosocial, physical, other impairment |
|  | SIS-16/ 3.0 | Stroke Impact Scale-16: 4 domains: strength, hand function, ADL/IADL, mobility; SIS 3.0 has 4 additional domains: communication, emotion, memory and thinking, participation/role function |
|  | SS-QoL-8/12 | the Stroke Specific Quality of Life Scale, 8 domains: activities, energy, mood, social roles, vision, language, thinking, personality,12 domains include extra mobility, upper extremity function, work and productivity, self-care |
|  | WHOQOL-BREF /100 | the World Health Organization Health Quality of Life assessment-BREF, 4 domains: physical health, psychological health, social relationships, environmental health; WHOQOL-100 has 2 additional domains: level of independence, spirituality |

Supplementary Table 4: Association between education and Health-Related Quality of Life among people with stroke: individual study results

| **Author (Year)** | **Define education** | **Unadjusted result** | **Adjusted**  **result** |
| --- | --- | --- | --- |
| Abubakar (2012) | years of formal education | – | X |
| Ali (2017) | illiterate, able to read and write, primary, intermediate, secondary, institute, college | ✓ | – |
| Alshahrani (2020) | no education, primary, high school, college | – | ✓ |
| Barbosa (2022) | elementary, middle, high school | – | ✓ |
| Baune (2006) | below secondary school, secondary school and above | ✓ | X |
| Butsing (2019) | no education, primary, secondary, bachelor, above | – | X |
| Choi-Kwon (2006) | years of education | X | – |
| Chou (2015) | years of education | – | X |
| Chuluunbaatar (2016) | less than college | X | – |
| Cramm (2012) | education level | X | X |
| Dayapoglu (2010) | illiterate, primary, high school | ✓ | – |
| Delcourt (2011) | level of education | ✓ | X |
| Dhamoon (2010) | at least high school education | – | X |
| Dianati (2021) | illiterate, primary, diploma, university | ✓ | ✓ |
| Gurcay (2009) | years of education | X | – |
| Heiberg (2020) | years of education | X | – |
| Huang (2010) | years of education | X | – |
| Jun (2015) | level of education | – | ✓ |
| Kariyawasam (2020) | primary, ordinary, advance | ✓ | ✓ |
| Kim (2021) | elementary, middle, high, college | X | – |
| Lourenço (2021) | years of education | ✓ | – |
| Mei (2022) | illiterate, primary, other | ✓ | – |
| Meyer (2010) | years of education | ✓ | X |
| (Continues with next page) | | | |
| **Author (Year)** | **Define education** | **Unadjusted result** | **Adjusted**  **result** |
| Ones (2005) | level of education | ✓ | – |
| Pucciarelli (2019) | level of education | ✓ | ✓ |
| Ramos-Lima (2018) | level of education | X | – |
| Salehi (2019) | level of education | – | X |
| Singhpoo (2012) | level of education | ✓ | – |
| Sok (2021) | level of education | ✓ | – |
| Szocs (2020) | years of education | X | – |
| Taufique (2016) | level of education | ✓ | ✓ |
| Tsalta-Mladenow (2021) | level of education | ✓ | – |
| Zemed (2021) | level of education | – | – |

✓: significant; X: not significant; –: ‘not reported’

Supplementary Table 5: Association between income indicator and Health-Related Quality of Life

among people with stroke: individual study results

| **Author (Year)** | **Define income** | **Unadjusted result** | **Adjusted result** |
| --- | --- | --- | --- |
| Alshahrani (2020) | monthly family income | – | ✓ |
| Barbosa (2022) | monthly patient income | – | X |
| Baune (2006) | monthly patient income | ✓ | X |
| Butsing (2019) | sufficient income | ✓ | ✓ |
| Choi-Kwon (2006) | monthly income | ✓ | ✓ |
| Delcourt (2011) | annual household income | ✓ | ✓ |
| Dianati (2021) | financial status | ✓ | X |
| Jun (2015) | monthly household income | – | ✓ |
| Kariyawasam (2020) | monthly income | ✓ | X |
| Kim (2021) | family monthly income | ✓ | ✓ |
| Lee (2015) | monthly household income | ✓ | – |
| Lourenço (2021) | family income | ✓ | X |
| Mei (2022) | monthly actual income | ✓ | ✓ |
| Meyer (2010) | income | X | – |
| Ramos-Lima (2018) | income | X | – |
| Salehi (2019) | with/without income | – | X |
| Singhpoo (2012) | household monthly income | X | – |
| Sok (2021) | monthly income | X | – |
| Zemed (2021) | income | – | – |

✓: significant; X: not significant; –: ‘not reported’

Supplementary Table 6: Association between occupation/work status and Health-Related Quality of Life among people with stroke: individual study results

| **Author (Year)** | **Occupation** | **Work status** | **Define indicator** | **Unadjusted result** | **Adjusted result** |
| --- | --- | --- | --- | --- | --- |
| Ali (2017) | X |  | employee, free job, retired, jobless, housewife | ✓ | – |
| Alshahrani (2020) |  | X | unemployed, employed | – | ✓ |
| Barbosa (2022) |  | X | retired, employed | – | X |
| Butsing (2019) |  | X | currently working | ✓ | ✓ |
| Choi-Kwon (2006) |  | X | unemployed, employed | ✓ | ✓ |
| Chou (2015) |  | X | employed | – | ✓ |
| Dayapoglu (2010) |  | X | housewife, retired, employed, other | ✓ | – |
| Delcourt (2011) |  | X | working or not | ✓ | X |
| Dianati (2021) |  | X | retired, self-employed, homemaker, worker | ✓ | X |
| Heiberg (2020) |  | X | working prior to stroke | X | – |
| Jun (2015) | X |  | Yes/No | – | ✓ |
| Kariyawasam (2020) | X |  | no occupation, business, with occupation | ✓ | X |
| Kim (2021) |  | X | employed | ✓ | – |
| Lourenço (2021) |  | X | retired | ✓ | – |
| Vincent-Onabajo (2015) |  | X | pre-stroke employment | ✓ | X |
| Paul (2005) | X |  | manual/nonmanual | ✓ | ✓ |
| Pedersen (2021) |  | X | working/student | – | X |
| Pucciarelli (2019) |  | X | unemployed | ✓ | – |
| Salehi (2019) | X |  | clerk/worker/housewife unemployed | – | X |
| Singhpoo (2012) |  | X | unemployed, employed | ✓ | – |
| Sok (2021) |  | X | Yes/No | ✓ | – |
| Sturm (2004) | X |  | skill level | ✓ | ✓ |
| Szocs |  | X | employment prior to stroke | X | – |
| (Continues with next page) | | | | | |
| **Author (Year)** | **Occupation** | **Work status** | **Define indicator** | **Unadjusted result** | **Adjusted result** |
| Tsalta-Mladenow (2021) |  | X | currently working | ✓ | – |
| Zemed (2021) | X |  | Private, housewife, farmer retired, government | – | – |
| ✓: significant; X: not significant; –: ‘not reported’ | | | | | |

Supplementary Table 7: Results by other socioeconomic status indicators and HRQoL among people with stroke: individual study results

| **Author (Year)** | **Other indicator** | **Define indicator** | **Significant in Unadjusted Analysis** | **Significant in Adjusted Analysis** |
| --- | --- | --- | --- | --- |
| Ali (2017) | residency | rural/urban | X | – |
| Alshahrani (2020) | residency | rural/urban | – | ✓ |
| Dayapoglu (2010) | residency | city/town/village | ✓ | – |
| Dayapoglu (2010) | health insurance | yes/no | X | – |
| Delcourt (2011) | health insurance | yes/no | X | – |
| Dhamoon (2010) | insurance status | with Medicaid | – | ✓ |
| Dianati (2021) | insurance type | social security, health, Relief Foundation, health service | ✓ | X |
| Ghotra (2018) | SES index | >= median score/<median score | X | ✓ |
| Jun (2015) | residency | rural/urban | – | ✓ |
| Szocs (2020) | neighbourhood | Poorest district/ other neighbourhood | X | – |

✓: significant; X: not significant; –: ‘not reported’

Supplementary Table 8: Sub-group analysis by indicators of SES and their associations with HRQoL with adjustment and non-adjustment of potential confounders

| **SES Indicator** | **adjustment** | **n** | **Estimate** | **95% CI** | **P** | **I^2^** | **P for subgroup** |
| --- | --- | --- | --- | --- | --- | --- | --- |
| **Income** | Yes | 2 | -0.06 | -0.16, 0.04 | 0.22 | 0 | 0.001 |
|  | No | 11 | -0.47 | -0.68, -0.25 | <0.0001 | 85.5% |  |
| **Education** | Yes | 2 | -0.10 | -0.17, -0.03 | 0.004 | 0 | 0.005 |
|  | No | 11 | -0.43 | -0.65, -0.21 | 0.0001 | 83.0% |  |

Supplementary Figure 1: Funnel plot for the global meta-analysis using combined SES indicators and overall HRQoL (n=17 studies)

**
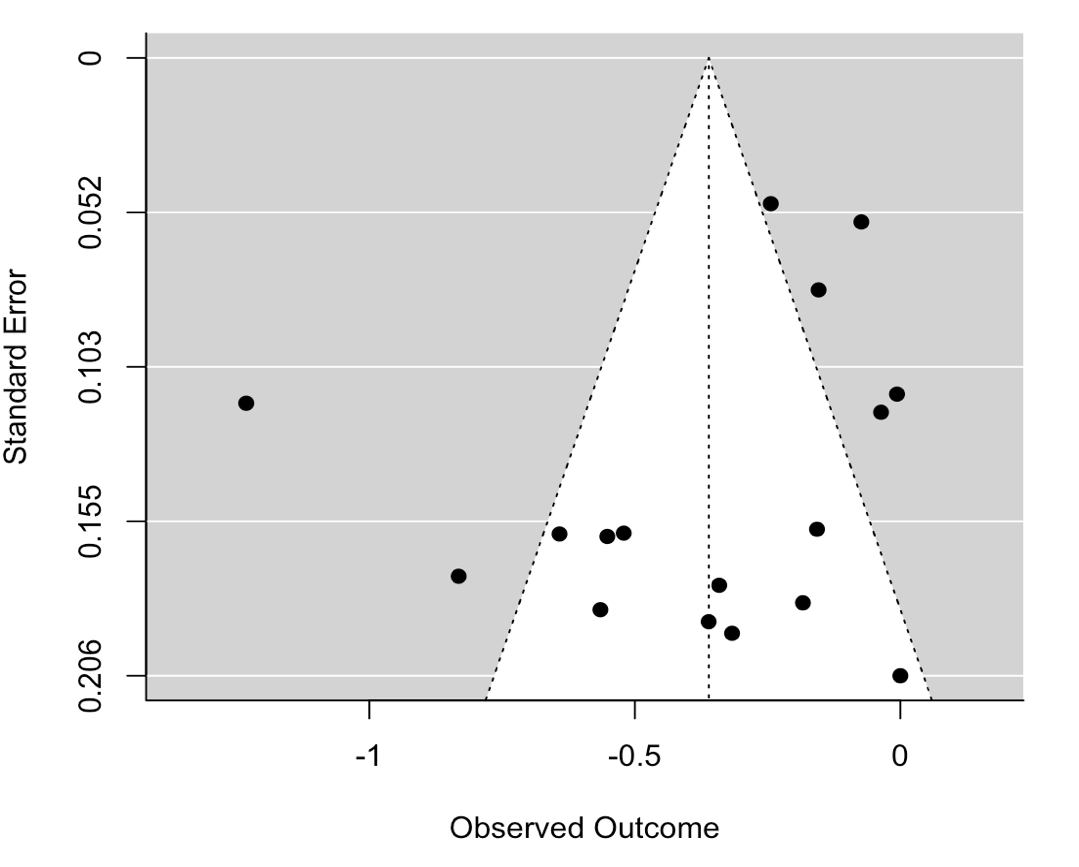
**
